# Use of Mineral Weathering Bacteria to Enhance Nutrient Availability in Crops: A Review

**DOI:** 10.3389/fpls.2020.590774

**Published:** 2020-12-11

**Authors:** Igor Daniel Alves Ribeiro, Camila Gazolla Volpiano, Luciano Kayser Vargas, Camille Eichelberger Granada, Bruno Brito Lisboa, Luciane Maria Pereira Passaglia

**Affiliations:** ^1^Departamento de Genética, Instituto de Biociências, Universidade Federal do Rio Grande do Sul, Porto Alegre, Brazil; ^2^Laboratório de Microbiologia Agrícola, Departamento de Diagnóstico e Pesquisa Agropecuária, Secretaria Estadual da Agricultura, Pecuária e Desenvolvimento Rural, Porto Alegre, Brazil; ^3^Programa de Pós-graduação em Biotecnologia, Universidade do Vale do Taquari – Univates, Lajeado, Brazil

**Keywords:** biological weathering, crushed rocks, plant growth-promotion, fertilizers, inoculants

## Abstract

Rock powders are low-cost potential sources of most of the nutrients required by higher plants for growth and development. However, slow dissolution rates of minerals represent an obstacle to the widespread use of rock powders in agriculture. Rhizosphere processes and biological weathering may further enhance mineral dissolution since the interaction between minerals, plants, and bacteria results in the release of macro- and micronutrients into the soil solution. Plants are important agents in this process acting directly in the mineral dissolution or sustaining a wide diversity of weathering microorganisms in the root environment. Meanwhile, root microorganisms promote mineral dissolution by producing complexing ligands (siderophores and organic acids), affecting the pH (via organic or inorganic acid production), or performing redox reactions. Besides that, a wide variety of rhizosphere bacteria and fungi could also promote plant development directly, synergistically contributing to the weathering activity performed by plants. The inoculation of weathering bacteria in soil or plants, especially combined with the use of crushed rocks, can increase soil fertility and improve crop production. This approach is more sustainable than conventional fertilization practices, which may contribute to reducing climate change linked to agricultural activity. Besides, it could decrease the dependency of developing countries on imported fertilizers, thus improving local development.

## Introduction

The green revolution in the 1950s caused a dramatic increase in food production. It was driven by the introduction of new technologies and practices, especially the massive use of synthetic fertilizers. The average percentage of yield attributable to commercial fertilizers ranged from 30–50% in the United Kingdom and United States, and tended to be much higher in the tropics (Stewart et al., 2005). In this context, the global demand for nitrogen (N), phosphorus (P), and potassium (K) is expected to reach 200,919 thousand tonnes by 2022 (FAO, 2019).

Despite the increase in crop production promoted in the last century by intensive farming and agrochemicals use, it has not been accomplished without environmental impacts (Ramankutty et al., 2018). The excess of high-soluble fertilizers has caused serious eutrophication in the aquatic environment (Bennett E.M. et al., 2001; Ghaly and Ramakrishnan, 2015). This phenomenon is responsible for the death of fish and benthic invertebrates due to the blooming of algae and cyanobacteria, causing a decrease in water quality through toxin production, hypoxia, and anoxia in the hydric bodies (Huisman et al., 2018).

Additionally, emissions of greenhouse gases (GHGs) represent the most important driver of human-induced climate change. The food production system is responsible for 29% of global GHG emissions, being the manufacture and application of fertilizers one of the main sources of emissions (Vermeulen et al., 2012). Even more concerning is the fact that climate change and agriculture are interrelated processes, i.e., the climate change will also affect agriculture. One of the most visible consequences of a warming world is an increase in the intensity and frequency of extreme weather events such as droughts, heatwaves, floods, and irregular patterns of precipitation. According to Vogel et al. (2019), climate extremes explain 18–43% of global maize (*Zea mays* L.), soybean (*Glycine max* L.), rice (*Oryza sativa* L.), and spring wheat (*Triticum aestivum* L.) crop yield variations. Warmer temperatures affect the plant’s ability to get and use moisture, thus impacting development and grain production (Cline, 2008). A two degrees Celsius warming could reduce major crop yields by 3–13% worldwide (Wang et al., 2020). As a consequence of global warming, drylands are also expected to expand, raising the risk of land degradation and desertification, consequently reducing areas available for crop production (Huang et al., 2017; Huang et al., 2020).

One of the major post-green revolution challenges is increasing food production to feed the growing human population and at the same time reduce the environmental impacts of crop production (Godfray et al., 2010; Cole et al., 2018). Responding to climate change demands intense adaptive and mitigating actions to ensure productivity (Jain, 2012; Springmann et al., 2018). Several alternatives for improving plant nutrition and consequently crop yield are applicable, such as the increase in efficiency of conventional fertilizer management considering a rational use of soil, water, and plant nutrient resources; the adoption of new technological fertilizers; development of genetically modified crops; and, the exploration of beneficial interactions between plants and rhizosphere microorganisms (Campbell et al., 2014; Bindraban et al., 2015; Timilsena et al., 2015; Gouda et al., 2018; Bailey-Serres et al., 2019).

Among the possible approaches to mitigate climate change, N-fixing plant growth-promoting rhizobacteria, collectively known as rhizobia, have been extensively investigated due to their exceptional quality to establish functional symbiosis with legumes (Lindström and Mousavi, 2020). As a result of this interaction, inoculants formulated with rhizobia have been successfully used for the reduction and replacement of N fertilizer in leguminous crops, especially considering soybean (Vargas et al., 2017). However, to develop bacterial inoculants that represent an efficient and viable alternative to fertilizers, the investigation of additional plant-bacteria associations characteristics is still in high demand (Franche et al., 2009; Sammauria et al., 2020). The rhizosphere bacteria have a huge potential for plant growth promotion (PGP) characteristics, such as the ability to secrete phytohormones and siderophores, increase availability and uptake macro- and micronutrients, trigger plant defense reactions against phytopathogens, and increasing plant tolerance to environmental stresses (Gouda et al., 2018).

Rock powders extracted from local reserves are also an important alternative to manufactured fertilizers. This strategy contributes to the recovery of degraded lands, the mineralogical rejuvenation of soils, and the increase in essential nutrients availability. Crushed rocks are less susceptible to nutrient leaching, besides being cheaper than conventional fertilizers (Fyfe et al., 2006; Manning and Theodoro, 2020). However, the recurrent low solubility of these materials limits its agronomic effectiveness as potential fertilizers. On the other hand, a key bacterial mechanism to improve plant nutrition relies on its capability to release nutrients as a result of the weathering process of soil minerals (Calvaruso et al., 2006). The action of such microorganisms could represent an ecologically correct and cost-effective way to improve not only the availability of indigenous nutrients from the soil but to also increase the solubilization of rock powders applied as a soil amendment.

In the present review, we discuss the potential of weathering bacteria to increase plant growth and crop yields by releasing macro- and micronutrients from soil minerals or crushed rocks applied in croplands.

## “Rocks for Crops”: An Eco-Friendly and Low-Cost Source of Nutrients for Plant Growth

The high cost of commercial fertilizers is a critical threat to food security in regions where small farmers play an important role in food production. This is, for instance, the case of Brazil, a country that imports about 70% of the nitrogen (N), 50% of phosphorus (P_2_O_5_), and more than 90% of the potassium (K_2_O) from total consumed fertilizers, so that international price fluctuations can prevent the purchase of fertilizers (Ogino et al., 2021). Consequently, the low fertilizer input on soils limits agricultural productivity, affecting the household earnings and diminishing dietary nutrients intake. There is a direct link between low soil fertility and chronic poverty in several countries, especially in Africa (Barrett and Bevis, 2015).

The “agrogeology,” or the use of “rocks for crops,” as Van Straaten (2002) defined, is a promising approach that could help countries to reduce the dependency on imported fertilizers using their own geological resources (Van Straaten, 2006). The domestic and unexplored resources of minerals are a low-cost source of plant nutrients for agricultural purposes. Despite the importance of such minerals being generally neglected due to their low price in the international market, they present a high potential for local development (Franks, 2020). In Brazil, the adoption of rock powder as a remineralizer has been a matter of intense and prolific agronomic research which resulted in a movement known as “rochagem” (Leonardos et al., 2000). The use and commercialization of such materials are regulated by law 12.890 (Brazil, 2013). Additionally, the Brazilian Ministry of Agriculture, Livestock, and Food Supply (MAPA), through normative instructions (IN 05 and 06), establishes clear definitions for registering and marketing of remineralizers. Those regulations ensure the agronomic effectiveness and security of commercial rock powder to farmers (Manning and Theodoro, 2020).

The application of rock powder is an advantageous practice adaptable to crop demands in different environments. However, remineralizers are especially beneficial in tropical soils, which are widely present in several developing nations. This type of soil is typically acid, susceptive to intense weathering rates, and has low N and P content. In this context, rock powder and minerals additionally help to raise the pH (liming effect), conserve nutrients, and preserve water quality (Leonardos et al., 2000; Van Straaten, 2002). Rocks fragments used as mulch material can reduce evaporative losses and conserve soil moisture (Groenevelt et al., 1989; Jiménez et al., 2017). Additionally, the usage of crushed rock is in conformance with organic agriculture requirements, a type of farming that is steadily increasing in the world (Manning and Theodoro, 2020).

The rocks successfully used in crop production include mostly single-nutrient rocks, such as rock phosphate (RP), and multi-nutrient silicate rocks (Van Straaten, 2006). RP is a raw material for the manufacturing of high-soluble P fertilizers and other industrial products. However, the direct application of RP in soils promotes a slow release of nutrients preventing losses by leaching, thus representing a more sustainable practice than conventional fertilization (Fayiga and Nwoke, 2016). The agronomical effectiveness of this approach depends on the chemical and mineralogical composition of these rocks, soil characteristics, environmental conditions, crop requirements, and agricultural management (Lompo et al., 2018). Considering that not all RP nutrients are readily available for plant uptake, solubilizing microorganisms could represent an alternative to increase its efficacy. The microorganisms could be inoculated in the soil or plants concomitantly with RP addition, or they could be used in the pretreatment of RP for partial solubilization before applying to the soil (Arcand and Schneider, 2006).

**FIGURE 1:**
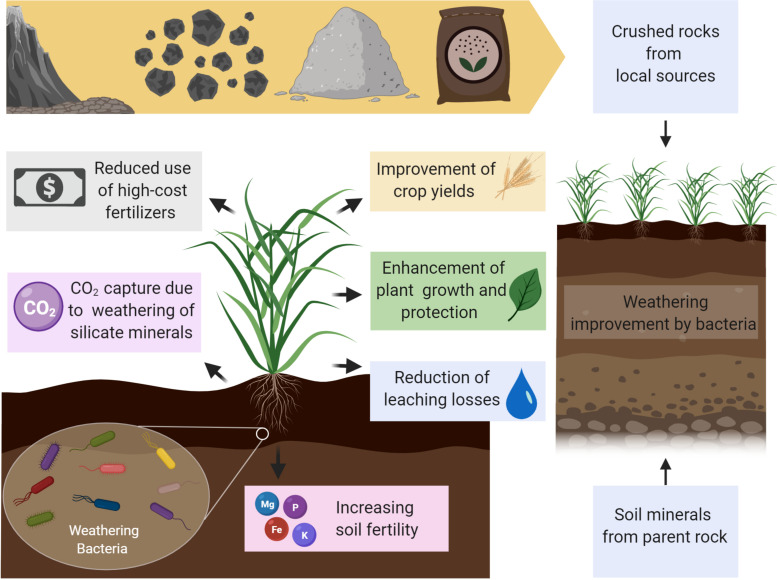
Beneficial effects for crop production from the use of rock powder associated with weathering bacteria. Rock powder extracted from local reserves is an important alternative to manufactured fertilizers. The inoculation of weathering bacteria increases plant growth and crop yields by releasing of macro- and micronutrients from soil minerals or crushed rocks applied in crop fields. Some weathering bacteria living in the rhizosphere can also display plant growth-promoting abilities (i.e., plant hormone production, N fixation, stress reduction, antimicrobials synthesis), enhancing plant development, or even protecting them against phytopathogens. The weathering of silicate rocks applied in croplands leads to the capture of CO_2_ and the release of carbonates and bicarbonates. This process can be microbiologically enhanced by weathering bacteria inoculated in soils (created with BioRender.com).

Agricultural production associated with inadequate management of soils and unbalanced fertilization results in nutrient depletion and yield reduction (Tan et al., 2005). The replenishment of leached or degraded soils to the nutrient levels of natural fertile soils can be achieved by supplying crushed rocks and silicate minerals of diverse composition into the system (Leonardos et al., 1987). Silicate rocks include a wide range of minerals (i.e., mica, quartz, pyroxene, and feldspar) and are a potential source of macro- and micronutrients. Despite being mostly explored as K sources, silicate can also supply other plant’s nutritional needs (Harley and Gilkes, 2000; Manning, 2010). However, in a similar way as RP, many types of silicate rocks are inappropriate for plant fertilization once the chemical and mineralogical properties of the rock must be adequate to soil characteristics and crop requirements. Even with a high absolute content of nutrients, the determinant aspect of nutrient availability is the rate of mineral dissolution (Manning and Theodoro, 2020). In this sense, the soil microbial community could also play a central role in that process increasing the benefits of silicate rocks (Figure 1).

Silicate rocks could act as remineralizers through the promotion of smectite (2:1 clays) minerals formation (Leonardos et al., 2000). Besides improving the chemical and nutritional properties of the soil, the application of such rocks and minerals also increases the soil CEC (cation exchange capacity) and suppresses toxic aluminum (Al) and manganese (Mn), present in high content in oxisols (Anda et al., 2015). In addition, the use of silicate rocks is a promising mechanism for GHG reduction considering that the weathering reactions of Ca/Mg-rich silicate minerals capture carbon dioxide from the environment and release bicarbonates (HCO_3_^–^) and carbonates (CO_3_^2–^). Such compounds could runoff to the sea, where they contribute to reducing ocean acidification (Andrews and Taylor, 2019). This acidification process is a result of increased atmospheric CO_2_ altering seawater carbonate chemistry, which affects mainly shell-forming organisms (Doney et al., 2009). Thus, the buffering effect of carbonate/bicarbonate released from rock weathering not only protects marine life but also promotes carbon storage for thousands of years in the oceans (Beerling et al., 2018).

## Biological Weathering: The Role of Weathering Microorganisms

Weathering is a term which describes the general process by which rocks are broken down into such things as sediments, clays, soils, and substances that are dissolved in water Zaharescu et al. (2020). This process is responsible for soil formation and it is one of the most relevant processes in the geological cycle of essential mineral elements, with singular importance in determining inherent fertility and availability of macro- and micronutrients that support the establishment and growth of plants (Wilson, 2004). The integrated effect of organisms, water, and atmosphere on dissolution and crystallization of minerals and rocks causes weathering to be often classified in physical, chemical, and biological processes (Formoso, 2006).

Thousands of years are usually needed to convert inorganic bedrock into fertile soils with distinct horizons (Kalev and Toor, 2018), being the initial process of soil formation highly dependent on microbial activity on weathering of bedrock material and formation of interfaces for nutrient turnover. The establishment of the initial microbial life is influenced by chemical composition and physical structure of the parent materials in which soils form, where compounds based on C or N are often scarce (Schulz et al., 2013).

Biofilm formation is a determinant step to the microbial establishment and weathering activity. Surface-attached bacteria are expected to promote a higher dissolution of elements from mineral particles than planktonic cells do (Ahmed and Holmström, 2015). Biofilms comport a matrix of extracellular polysaccharide, forming a hydrated gel that wraps microorganisms and adheres them to rock surfaces. This environment supports and protects microbial activity mostly against desiccation and external interferents (Kemmling et al., 2004). Besides that, biofilms allow concentrating organic acids, siderophores and other chelating compounds, and other weathering agents in the organism/mineral interface. It also favors cell-to-cell communication and microbial interactions. Consequently, this structure allows a synergic effect of different microorganisms and biological processes on mineral dissolution (Finlay et al., 2020).

Lichens are one of the pioneer organisms that establish on rocks. The adhesion of lichen causes physical fragmentation of minerals by hyphal penetration in rock surfaces (Banfield et al., 1999; Chen et al., 2000). The fixed carbon provided by the photosynthetic symbiont promotes the development of fungi and other microorganisms. The subsequent secretion of several organic acids causes an intense chemical weathering process (Adamo and Violante, 2000). Several other organisms could also be powerful agents in the initial weathering of rocks, including fungi, bacteria, cyanobacteria, archaea, and mosses. Those microorganisms cause mineral disaggregation, dissolution, hydration, and secondary mineral formation directly or indirectly (Jackson, 2015; Zaharescu et al., 2020).

Plant nutrients, except for N, derive ultimately from the weathering of primary minerals. Therefore, the action of pioneer microorganisms is essential for the subsequent settlement of plants. Plants, in their turn, also promote a significant weathering of soil minerals through improving basic soil properties, affecting water dynamics and cycling of cations in soil solution (Li et al., 2006). Root exudates contain organic acids and chemical ligands that promote mineral solubilization (Kelly et al., 1998; Lucas, 2001). The exudation rates occur in response to plant nutrition demands and rise with increasing root surface area and tip number (Aoki et al., 2012). Besides that, plants also release sugars, amino acids, enzymes, fatty acids, sterols, growth factors, vitamins, and secondary metabolites, transforming the region surrounding the roots in a nutritional environment that could support a luxuriant diversity of microorganisms (Vives-Peris et al., 2020). That region, called rhizosphere, promotes an intense weathering of soil minerals at higher rates than those of bulk soil (Richter et al., 2007).

**FIGURE 2:**
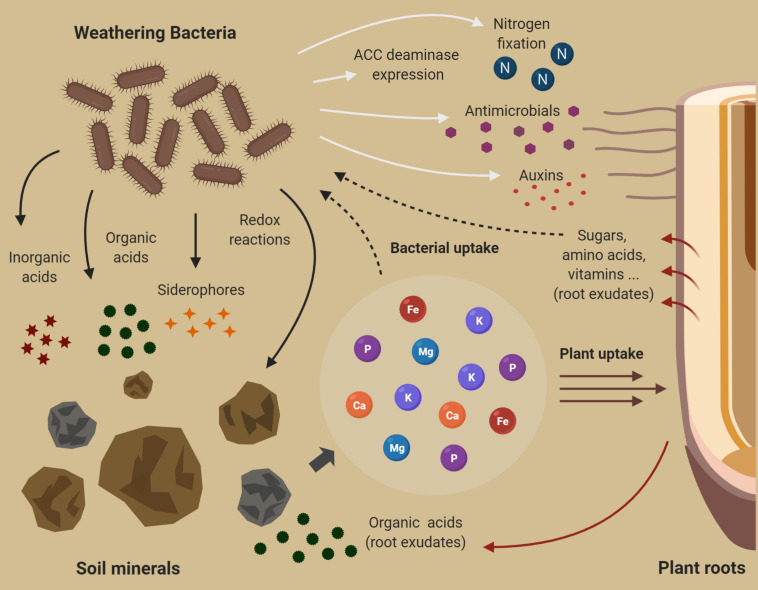
Mechanisms related to weathering of rocks and minerals by soil bacteria. Weathering bacteria perform redox reactions or produce compounds such as organic/inorganic acids and chelating agents that directly affect the dissolution of minerals (represented by curved black arrows). Rhizosphere bacteria can promote plant growth by different mechanisms, such as plant hormone production, N fixation, stress reduction, antimicrobials synthesis (represented by white arrows), and this way, these microorganisms increase the mineral weathering performed by plants indirectly. The beneficial effect of rhizobacteria enhances plant root development and consequently the releasing of exudates (i.e., organic acids) related to weathering by plants. Different organic compound of root exudates (represented by red arrows) supplies bacterial growth and indirectly favors nutrients release from minerals by weathering bacteria activity. Dashed arrows represent nutrients and organic compound uptake by bacteria (created with BioRender.com).

Rhizosphere bacteria can directly solubilize minerals from soil (direct microbial weathering) or can increase plant fitness and growth, benefiting from the plant weathering activity (indirect microbial weathering) (Figure 2). As previously mentioned, different diazotrophic bacteria colonize the rhizosphere and supply N compounds via the N fixation process (Goswami et al., 2016), being such ability especially important for the weathering activity of pioneer plants in the early stage of soil formation when N is frequently scarce (Schulz et al., 2013). Interestingly, roots development and architecture can also be directly modulated by associated bacteria (Verbon and Liberman, 2016). These organisms could affect the balance of phytohormones by the synthesis of auxins. Such compounds increase the growth of root hairs and lateral roots, enhancing root exudation and consequently increase the mineral weathering performed by plants (Calvaruso et al., 2006; Spaepen and Vanderleyden, 2011).

The expression of 1-aminocyclopropane-1-carboxylate (ACC) deaminase is also an important bacterial mechanism for plant growth promotion. This enzyme regulates the ethylene levels on plants, reducing the stress response under different biotic and abiotic conditions (Glick, 2014). Rock weathering in dry environments and deserts, for example, can be mediated by the action of plant and rhizosphere microbes (Puente et al., 2004a,b). Such microorganisms exhibit the ability of plant growth promotion, favoring plant colonization in these extreme conditions.

Rhizosphere microorganisms can also inhibit the development of plant pathogens by the production of enzymes, antimicrobial peptides, antibiotics, antifungals, and other antimicrobial compounds (Tariq et al., 2017; Volpiano et al., 2018). Plant and associated bacteria keep an important chemical communication at the rhizosphere level (Rosier et al., 2018). The “cry for help” model establishes that stressed plants assemble protective microbiomes recruiting beneficial bacterial through chemotactic metabolites in root exudates (Rolfe et al., 2019). These close interactions with bacteria are strategic for plant adaption to different environments and consequently can affect plant weathering activity.

## Direct Mechanisms of Mineral Weathering by Bacteria: A General Overview

The mineral dissolution resulting from the microbial activity can be either an active or a collateral process. The weathering outcome could be a consequence of diverse energy-consuming processes that release metabolic by-products that can affect mineral solubilization (Banfield et al., 1999, Bennett P.C. et al., 2001). Nevertheless, weathering frequently occurs under cellular control in response to microorganism’s nutrition and growth requirements. Therefore, the mineral composition and the demands of the microbial community directly influence weathering rates (Bennett P.C. et al., 2001). The main mechanisms usually related to bacterial weathering include pH changes surrounding the mineral particle and proton promoted dissolution, chelation of elements present in a mineral matrix or the soil, and redox reactions (Figure 2; Samuels et al., 2020).

Different bacterial processes result in inorganic acid production. Sulfuric acid, for example, can be generated by sulfur-oxidizing bacteria (i.e., some species belonging to the *Thiobacillus* genus), while nitrifying bacteria can produce nitric acid during nitrification reactions (Ranalli et al., 2019). The pH change promoted by microbial activity can alter the solubility of ions, perturbing the ionic concentration equilibrium in the mineral-solution interface by directing the equilibrium in favor of mineral dissolution. The acidification process is frequently related to organic or inorganic acid production. Some of the most frequent organic acids include formic, citric, gluconic, acetic, lactic, oxalic, succinic, and pyruvic acids. These substances are usually byproducts of the metabolism of carbon sources (Zhu et al., 2014; Samuels et al., 2020).

Carbonic acid formation as a result of CO_2_ releasing from aerobic respiration also represents an important mechanism related to acidification. However, this process is a relatively slow reaction that may be enhanced by the expression of carbonic anhydrase, which catalyzes the reversible hydration of CO_2_ (CO_2_ + H_2_O ⇌ HCO_3_^–^ + H^+^; Tripp et al., 2001). This enzyme is widespread and present in several bacterial strains (Smith and Ferry, 2000), with a relevant function in mineral weathering such as evidenced for calcite dissolution (Xiao et al., 2014).

Siderophores and organic acids are the main chelating agents produced by weathering bacteria. Organic acids not only acidify the mineral surrounding environment, but the deprotonated forms of these compounds also chelate ions in soil solution, affecting dissolution rates. The carboxylic groups of organic acid structures, dissociated from H^+^ and negatively charged, are ligand sites for cations. The number of carboxyl, therefore, affects the chelating capacity, and tri- and dicarboxylic acids are more efficient solubilizers than monocarboxylic acids (Adeleke et al., 2017; Lazo et al., 2017). Siderophores are ferric ion-specific chelators secreted under iron stressed condition. These non-ribosomal peptides have been classified as catecholate, hydroxamate, carboxylate, and mixed types (Khan et al., 2018). These substances are especially relevant in the solubilization of Fe silicates and other Fe-bearing minerals (Ahmed and Holmström, 2014; Torres et al., 2014). Conveniently, siderophores can non-specifically complex different other metallic ions (i.g. Co, Cr, Mn, and Mo) by acting on the weathering of rocks and minerals containing these elements (Duckworth and Sposito, 2005; Liermann et al., 2005; Bi et al., 2010; Duckworth et al., 2014).

Oxidation and reduction reactions of a compound present in a mineral particle are expected to destabilize the crystalline structure, causing its dissolution (Uroz et al., 2009). Several bacterial strains use metals as terminal electron acceptors during anaerobic respiration. A well-known example is Fe-reducing bacteria, such as members from the genera *Desulfuromonas* (Vandieken et al., 2006) and *Shewanella* (Bose et al., 2009). These bacteria can use Fe (III) from different Fe oxides and clay minerals (Colombo et al., 2014). An Additional important weathering process is related to Fe oxidation reactions. *Geoalkalibacter ferrihydriticus* Z-0531^T^, for example, can anaerobically oxidizes Fe^2+^ with carbonate as an electron acceptor. This reaction causes the weathering of phyllosilicates, such as biotite and glauconite (Zavarzina et al., 2016).

## Efficacy and Specific Mechanisms of Weathering Bacteria to Enhance Nutrient Availability to Crops

Several studies have reported an enhanced release of key nutrients from minerals and rocks due to the inoculation of efficient weathering bacteria on plants and/or soils (Tables 1, 2). Some studies have evaluated how the plant-bacteria interaction increases the dissolution of a range of elemental nutrients (Puente et al., 2004a,b; Calvaruso et al., 2006; Lopez and Bacilio, 2020). In the present review, we initially focus on reports addressing the weathering of P- and K-bearing rocks, the two main macronutrients from mineral sources.

**TABLE 1:** Examples of PSB inoculation associated with rock phosphate fertilization.

**Bacterial species**	**Crop/plant specie**	**Mineral source**	**Type of experiment**	**Results**	**References**
*Burkholderia gladioli* MTCC 10216; *B. gladioli* MTCC10217, *Enterobacter aerogenes* MTCC 10208, and *Serratia marcescens* MTCC 10238	*Stevia rebaudiana*	Mussoorie rock phosphate	Pot experiment	Enhanced plant growth and content of stevioside and rebaudioside-A metabolites. Increased P availability in soils	Gupta et al., 2011
*Staphylococcus scirui; Bacillus pumilus; Bacillus subtilis*; and *Bacillus cereus*	Rice	Rock phosphate*	Pot/field experiment	Increased yield	Rajapaksha and Senanayake, 2011
*Pseudomonas* sp. RT5RP2 and *Pseudomonas* sp. RT6RP	Lentil (*Lens culinaris* Medik. cv. VL Masoor 507)	Udaipur rock phosphate	Pot experiment	Increased P uptake by plants	Selvakumar et al., 2013
*Pantoea cypripedii* (PSB-3) and *Pseudomonas plecoglossicida* (PSB-5)	Wheat (var. PBW-621) Maize (var. DKC-9106)	Rock phosphate*	Field experiment	Increased the crop growth, biomass, grain yield, and total P uptake by plants	Kaur and Reddy, 2015
*Burkholderia* sp. UFLA 04-21; *Paenibacillus kribbensis* UFLA 03- 10; *Enterobacter* sp. UFPI B5-6; and *Pseudomonas* sp. UFPI B5-8A	Rice	Bayóvar rock phosphate	Pot experiment	Increased plant biomass, number of tillers and accumulation of nutrients	Costa et al., 2015
*Bacillus thuringiensis* “serovar *ostriniae*” (PSM1) and *Bacillus* sp. Cp-h60 (PSM2)	Chickpea (*Cicer arietinum* L.) var. Noor-2009 and lentil var. PunjabMasoor-2009	Rock phosphate*	Pot experiment	Increased soil aggregate stability and P-release. Increased plant growth, dry weight of nodules, and grain yield	Ditta et al., 2018
*Pseudomonas corrugata* SP77, *Pseudomonas koreensis* LT62, and *Pseudomonas frederiksbergensis* G62	*Medicago truncatula* Gaertn.	Tunisian rock phosphate	Pot experiment	Enhanced shoot dry weight and nodule fresh weigh	Ben Zineb et al., 2020
*Bacillus* sp. SM0307, *Bacillus* sp. SS0303, *Bacillus* sp. SS0306, *Bacillus* sp. RP10, and *Bacillus* sp. RP5.	Wheat var. FARAJ	Moroccan natural phosphate	Glass tubes culture	Improved biometric parameters and P content of plants	Azaroual et al., 2020
*Serratia plymuthica* BMA1	*Vicia faba* L. (var. bachar)	Rock phosphate*	Pot experiment	Increased plant growth and P uptake by plants	Borgi et al., 2020

*Rock phosphate origin not specified.

**TABLE 2:** Examples of plant inoculation with KSB associated to fertilization with K-bearing rocks/mineral.

**Bacteria**	**Crop**	**Mineral source**	**Type of experiment**	**Results**	**References**
*Bacillus cereus*	Potato (*Kara* Spp.)	K-feldspar	Field experiment	Increased plant growth, yield, soil nutrient availability, and plant nutrient uptake	Ali et al., 2020
*Klebsiella oxytoca* KSB-17	Maize	Waste mica	Pot experiment	Improved plant growth	Imran et al., 2020
*Pseudomonas* sp.	Tomato (*Solanum lycopersicum* L.)	Muscovite	Pot experiment	Increased plant biomass and K uptake	Sarikhani et al., 2018
*Bacillus pseudomycoides* O-5	*Camellia sinensis* L.	Waste mica	Pot experiment	Increased K availability in soil, and that in turn facilitated K uptake by plant	Pramanik et al., 2019
*Bacillus subtilis* ANctcri3; *B. megaterium* ANctcri7	*Amorphophallus paeoniifolius* (Dennst.) Nicolson (elephant foot yam)	Feldspar rock powder (K 3.9%)	Field experiment	Increased tuber yield	Anjanadevi et al., 2016
*Enterobacter* sp. GL7, *Klebsiella* sp. JM3, *Klebsiella* sp. XF4, and *Klebsiella* sp. XF11	Tabaco	K-feldspar	Pot experiment	Increased plant biomass and plant uptake of K and N	Zhang and Kong, 2014
*Bacillus mucilaginosus, Azotobacter chroococcum*, and *Rhizobium* spp.	Maize cv. “Navjot” and wheat cv. “HD-2733”	Waste mica	Hydroponic experiment	Increased biomass accumulation and K uptake by plants as well as chlorophyll and crude protein content in plant tissue	Singh et al., 2010
*Bacillus mucilaginosus*	Sudan grass (*Sorghum vulgare* Pers.) var. Sudanensis	Waste mica	Pot experiment	Increased biomass yield and K uptake	Basak and Biswas, 2009
*Bacillus edaphicus* NBT	Cotton (*Gossypium hirsutum* L.) cv. Simian and rape (*Brassica napus* L.) cv. Zhongyou-1	Illite	Pot experiment	Increased plant growth, K uptake by plants and K availability in soil	Sheng and He, 2006

### Weathering Bacteria and P Availability

Several studies have explored the action of weathering bacteria of releasing nutrients from minerals and rocks, being the solubilization of P the best characterized. This is not a coincidence, considering that P is the second most important nutrient required for the maximum yield of agriculturally important crops. P can be found in organic and inorganic forms in the soil, but a small percentage is presented in a soluble form rapidly available to plant uptake (Syers et al., 2008). Even when soluble forms of inorganic P fertilizers are applied to the soil, they are rapidly immobilized due to complex formation with highly reactive Fe and Al oxides in acid soils and with calcium in calcareous soils (Chacon et al., 2006).

A specific group of bacteria known as P-solubilizing bacteria (PSB) has been reported as capable of solubilizing inorganic P, including strains belonging to species from the genera *Rhizobium* (Sridevi and Mallaiah, 2009), *Bacillus* (Saeid et al., 2018), *Methylobacterium* (Agafonova et al., 2013), *Pantoea* (Son et al., 2006), *Enterobacter* (Mendoza-Arroyo et al., 2020), *Bradyrhizobium* (Marra et al., 2011), *Gluconacetobacter* (Crespo et al., 2011), *Azospirillum* (Rodriguez et al., 2004), *Microbacterium* (Rivas et al., 2004), *Paenibacillus* (Zhang et al., 2013), *Burkholderia*, *Pseudomonas*, and *Streptomyces* (Zhao et al., 2014).

The use of PSB was first reported in the 1950s, when “phosphobacterin,” a fertilizer consisting of kaolin rocks impregnated with spores of a *Bacillus megaterium* var. *phosphaticum* (formerly *Megatherium viphosphateum*) strain was used in soils in the URSS, resulting in crop yield increases ranging from 0 to 70% (Cooper, 1959; Menkina, 1963). Since the first reports of 70 years ago, PSB benefits have been demonstrated for different and agronomically relevant crops. The main observed effects in crop production include the reduction in the application of traditional fertilizers (Sundara et al., 2002), the improvement on the efficacy of low-cost RP, the reduction of P losses in the soil, and the improvement of plant development (Kaleem Abbasi and Manzoor, 2018). In Table 1, different studies were summarized considering the concomitant effect of PSB inoculation with the RP soil amendment.

P-solubilizing bacteria can act synergistically with other plant growth-promoting microorganisms, enhancing plant fitness and plant nutrition at higher rates than those arising from a single factor (Yu et al., 2012; Zaidi et al., 2017). One of the most frequent strategies for this is to combine PSB with N-fixing symbiotic bacteria for inoculation of legumes. The co-inoculation of *Bradyrhizobium* with distinct *Pseudomonas* PSB strains on soybean can enhance nodule number, plant growth, grain yield, P uptake by plants and reduce the P fertilizer input (Son et al., 2007; Afzal et al., 2010; Argaw, 2012). Similar effects have also been reported by combining other rhizobia with different PSB strains, such as co-inoculation of diazotrophic *Rhizobium* sp. and PSB *Pseudomonas fluorescens* on common bean (Samavat et al., 2012), *Bradyrhizobium* sp. and PSB *Pantoea* sp. on peanut (Taurian et al., 2013), *Sinorhizobium meliloti* B399 and the PSB strains *Pseudomonas* sp. FM7d and *Bacillus* sp. on alfalfa (Guiñazú et al., 2010), and *Mesorhizobium* sp. and PSB *Bacillus* sp. on chickpea (Wani et al., 2007). Another promising approach is the combination of PSB with mycorrhizal fungi. While PSB enhance the P availability from sparingly soluble P sources, fungus efficiently absorbs and transports the nutrient to the host plant. This strategy has been reported to significantly increase plant development and P uptake on different plant species and soil conditions (Azcon et al., 1976; Suri and Choudhary, 2013; Nacoon et al., 2020). Triple inoculation of PSB, mycorrhizal and diazotrophic bacteria are also a potential alternative to enhance the efficiency of RP fertilization. Zaidi and Khan (2006) reported an improvement in plant vigor, nutrient uptake, and yield of green gram fertilized with RP and inoculated with N-fixing *Bradyrhizobium* sp., PSB *Bacillus subtilis* and mycorrhizal *Glomus fasciculatum.*

The mechanisms by which P is released from insoluble sources by PSB could be mediated by acidification, chelation, exchange reactions, and formation of exopolysaccharide (EPS), which may be related to the production of organic acids (Chen et al., 2016; Wei et al., 2018) or the release of protons accompanying respiration or NH_4_^+^ assimilation (Illmer and Schinner, 1995). EPS act synergically with organic acids on P solubilization. These polymeric substances change the homeostasis of P-solubilization by displaying a P-holding capacity, pushing it toward dissolved P, consequently resulting in greater P release from insoluble sources (Yi et al., 2008).

Gluconic acid is a frequently reported organic acid agent produced by PSB associated with P solubilization. Rodriguez et al. (2004) evaluated the *Azospirillum brasilense* strains Cd and 8-I and *Azospirillum lipoferum* JA4 for P solubilization from sparingly soluble calcium phosphate. For the three PSB strains, gluconic acid was the sole organic acid detected by HPLC. As expected, the released soluble P from calcium phosphate was reported as associated with a reduction in the pH of the medium. Similarly, Kim et al. (1997) related an increase in soluble P concentrations associated with a decrease in the pH of the culture medium while evaluating ISL19, a *Rahnella aquatilis* PSB strain isolated from the soybean rhizosphere that shows a strong ability to solubilize hydroxyapatite. In this study, gluconic acid was also the main organic acid released into the medium by the bacteria.

The conversion of glucose to gluconic acid by PSB is catalyzed by glucose dehydrogenase (GDH), an important membrane-bound enzyme involved in the direct oxidative pathway of glucose catabolism (Olijve and Kok, 1979; Neijssel et al., 1983). Genetic engineering approach with genes encoding glucose dehydrogenase (*gcd*) has proved that mineral phosphate solubilization by PSB was accompanied by gluconic acid formation. De Werra et al. (2009) assessed the role of gluconic acid production for P-solubilizing ability of *P. fluorescens* CHA0. The authors used engineered CHA0 mutants with deletions of *gcd* and gluconate dehydrogenase (*gad*), the gene required for the conversion of gluconic acid into 2-ketogluconate. The wild-type CHA0 and the Δ*gad* mutant (CHA1197) strongly acidified the medium, while the absence of *gcd* in the Δ*gcd* (CHA1196) and the Δ*gcd* Δ*gad* (CHA1198) mutants resulted in less acidification of the glucose solution. The wild-type CHA0 produced acid on tricalcium phosphate [Ca_3_(PO_4_)_2_] medium, which resulted in the formation of a clear halo (4.2 ± 0.8 mm in diameter). The *gcd* defect in the strains CHA1196 and CHA1198 resulted in a loss of P-solubilizing ability, which resulted in no halo formation. The absence of *gad* caused the accumulation of gluconic acid in the strain CHA1197, which resulted in a significantly greater P solubilization (halo diameter, 5.6 ± 0.9 mm). Several other studies characterized the role of GDH on P solubilization by different bacterial species, such as *Erwinia herbicola* (Goldstein and Liu, 1987), *Pseudomonas frederiksbergensis* (Zeng et al., 2016), and *Serratia marcescens* (Krishnaraj and Goldstein, 2001).

### Weathering Bacteria and K Availability

After P and N, K is often considered the most important nutrient affecting the growth, metabolism, and development of plants. Among the essential elements, K is usually the most abundant in soils. This nutrient can be found as water-soluble or solution K directly available for plant uptake; exchangeable K on the surface of clay minerals and organic matter, which is easily released to replenish water-soluble K; non-exchangeable K mainly on silicate layers and slowly released; and, insoluble rocks and minerals containing K, which represent more than 98% of the soil K and can only become available slowly through long-term soil weathering. Consequently, only 0.1–2% of the K content in soils is easily available for plant uptake (Reitemeier, 1951; Sparks and Huang, 1985).

Soluble K-fertilizers based on potash (“potassium-rich salt”) are produced mainly from mined salts (i.e., sylvite and carnallite). Comparing to N and P fertilizers, these sources demand less industrial and chemical processing to result in a fertilizer product (Manning, 2018). The main resources of K salts are in the northern hemisphere. Canada, Russia, and Belarus produced more than 90% of world potash (Ciceri et al., 2015). Historically, access to conventional potash fertilizer could be difficult due to high prices, transport, and even geopolitical reasons. This situation leads to the innovative use of different materials and geological resources as K fertilizers, being the use of silicate rocks a clear example of this (Manning, 2018). Despite the low solubility of silicate rocks, a group of weathering bacteria known as K-solubilizing bacteria (KSB) display an efficient ability to release K and others plant nutrients from these sources (Etesami et al., 2017).

Several works have isolated plant-associated KBS and characterized their ability to solubilize K-bearing minerals *in vitro*. Interestingly, these KSB are also reported with additional plant growth-promoting traits (i.e., N-fixation, P solubilization, auxin production), resulting in plant growth and nutrition increase under greenhouse and field conditions (Sugumaran and Janarthanam, 2007; Sangeeth et al., 2012; Bakhshandeh et al., 2017; Xiao et al., 2017; Yaghoubi Khanghahi et al., 2018; Sun et al., 2020). Despite the plant beneficial effect of these bacteria, frequently their ability to increase the solubility of rock powder or minerals applied as fertilizers in soils remains uncharacterized. Studies reporting increased effectiveness of fertilization from K-bearing minerals associated with inoculation of KSB are summarized in Table 2. Such examples demonstrate the potential of weathering bacteria to stimulate the use of rock powders as efficient fertilizers for different crops.

Similarly to PSB co-inoculation strategies, KSB could be employed with N-fixing bacteria, PSB, and mycorrhiza, displaying an increased effect on plant growth-promoting, soil nutrient availability, and nutrient uptake by plants (Han et al., 2006; Basak and Biswas, 2010; Dominguez-Nuñez et al., 2016). The weathering of K-bearing minerals also occurs by similar mechanisms described to P-solubilization, i.e., lowering soil pH, acidolysis, exchange reactions, complexation, and organic acid production (Sattar et al., 2019). The organic acid release seems to be the most widely studied mechanism of K solubilization. Several acids are reported, such as acid citric, ferulic, coumaric, malic, syringic (Setiawati and Mutmainnah, 2016). The common mechanism of gluconic acid production via direct oxidation of glucose by GDH is also related to K-solubilization (Wagh et al., 2016).

### Weathering Bacteria and the Availability of Multi-Nutrients and Other Beneficial Elements

Silicate rocks can be used not only as K sources but it could also supply additional plant nutrition needs as it contains minor amounts of other macro and micronutrients (Priyono and Gilkes, 2004; Van Straaten, 2006). The soil inoculation with KSB and PSB could increase the plant uptake and biomass content of diverse nutrients (Costa et al., 2015; Yaghoubi Khanghahi et al., 2018). The combination of different rocks or mineral resources with these weathering bacteria represent an important strategy to supply plants with multi-nutrient, especially using K-bearing rocks concomitant with RP fertilizers. This approach was adopted by Leyval and Berthelin (1989) using a mixture of sand, RP, and mica (phlogopite) as the only source of P, Fe, Mg, and Al to cultivate *Fagus sylvatica* (common beech). Plants were inoculated with mycorrhizal fungus *Laccaria laccata* and an efficient P-solubilizing *Agrobacterium* strain. After two years, the co-inoculation treatments increased nutrients uptake and dry matter of roots. Using biotite and anorthite mixed into silica sand as sources of nutrients, Balogh-Brunstad et al. (2008) evaluated the effects of weathering bacteria combined with mycorrhizal fungi on chemical weathering for pine growth. The fungi *Pisolithus tinctorius* and *Suillus tomentosus* in association with bacteria (*Ewingella americana*, *B. megaterium*, and *Pantoea agglomerans*) were able to increase weathering fluxes, plant biomass, and cation uptake (Ca^+2^, Mg^+2^, and K^+^).

Positive effects of combining RP and K-bearing minerals were also reported by Badr et al. (2006). Six bacterial strains isolated from feldspar samples were capable of dissolve silicate minerals and RP in different *in vitro* growth conditions. Bacterial inoculation combined with the addition of K and P-bearing minerals on sorghum increased the dry matter of plants by 48–58% in different types of soil. The P uptake by plants increased 41–93%, while the K uptake increased 71–116%. Similarly, Abou-el-Seoud and Abdel-Megeed (2012) observed the highest improvement in maize growth and nutrient uptake when PSB (*B. megaterium* var. *phosphaticum* strain) and KSB (*Bacillus mucilaginosus* and *B. subtilis* strains) were co-inoculated in conjunction with direct application of rock phosphate (apatite) and K materials (feldspar and illite powder). Han et al. (2006) also investigated the potential of *B. megaterium* var. *phosphaticum* PSB and *B. mucilaginosus* KSB to increase the growth of pepper and cucumber. The highest nutrient availability and uptake also occurred when PSB and KSB were co-inoculated with RP and illite powder.

The supply of macro- and micronutrients is not the only advantage resulting from the weathering of silicate rocks. Other released elements, such as silicon (Si), can be important for plant health. Si is the second most abundant element in the Earth’s crust after oxygen. Si is traditionally not considered essential for plant development, however, it is found in plants at concentrations ranging from a fraction of 0.1 to 10% of dry weight (Epstein, 1994). Recently, Si has gained increasing attention in agriculture because of the accumulating evidence of its beneficial effects on several crop species. For further information on the Si role on plant growth and resistance to biotic and abiotic stresses, please see Currie and Perry (2007) and Luyckx et al. (2017).

The addition of Si fertilizer may become a recurrent agricultural practice in the future considering that the Si in agricultural soils is depleting due to its continual removal with the harvested product (Keller et al., 2012; Haynes, 2014). Recently, Kang et al. (2017) demonstrated the ability of the rice root-associated rhizobacteria *Burkholderia eburnean* CS4-2 to solubilize silicate and promote Si uptake in plants. Under greenhouse conditions, soil inoculation with CS4-2 combined with insoluble silica fertilization significantly promoted the growth of rice plants. Similarly, Chandrakala et al. (2019) showed the potential of *Rhizobium* sp. IIRR-1 to release soluble silica from insoluble inorganic (Ca, Al, K, and Mg) silicates. The Si solubilizing isolate also produced IAA and showed ACC deaminase activity *in vitro*. The IIRR-1 strain was inoculated in rice seeds under gnotobiotic conditions and showed the capacity to colonize plant roots and increase seedling vigor by 29.18%. Interestingly, Hu et al. (2018) isolated different Si-solubilizing bacteria from the gut of the earthworm *Pheretima guillelmi*. Three selected strains (*Flavobacterium* spp. 3C1, *Pseudomonas* spp. 3C5, and *Bacillus* spp. 4A2) were able to release soluble Si from feldspar and quartz powder. When inoculated in maize, the *Flavobacterium* spp. 3C1 increased Si uptake by plants and increased soluble Si contents in the soil.

## Concomitant Use of Rocks and Weathering Bacteria as Fertilizers: Challenges and Future Directions

Concerning the interaction between rocks and microorganisms, environmental variations interfere not only with physical and chemical weathering rates of minerals but also with microbial survival, distribution, and metabolism, consequently with microbial weathering activity (Robert and Berthelin, 1986; Gordon, 2005; Gleeson et al., 2016). Different soil types, climate conditions, indigenous microbial communities, plant genotype, crop requirements, and farming practices strongly affect the plant growth-promoting effects of bacteria. These factors must be carefully investigated before the recommendation of new strains as inoculants (Saad et al., 2020). Several approaches have been developed and adopted to increase microbial inoculants’ effectiveness and can be suitable to improve the use of weathering bacteria associated with rock fertilizers.

*In vitro* investigations of microbial weathering have demonstrated that the solubilizing ability of PSB and KSB is mainly affected by factors such as the concentration of soluble P or K, C and N sources, and pH (Parmar and Sindhu, 2013; Musarrat and Khan, 2014). In the same way, soil experiments and field trials indicate that N fertilization, organic amendment, and lime addiction directly influence the effectiveness or abundance of solubilizing organisms (Adnan et al., 2017; Zheng et al., 2017; Kaleem Abbasi and Manzoor, 2018; Zheng et al., 2019). Therefore, these factors can be modulated to enhance bacterial solubilization of minerals. As an example, Mpanga et al. (2019) demonstrated that stabilized ammonium fertilizers increased the RP solubilization by different PSB inoculants better than using nitrate fertilizers. Likewise, organic amendments, such as manure and vermicompost, can significantly increase the bacterial efficiency of solubilizing P and K-bearing rocks in soils (Baldotto et al., 2012; Abbasi et al., 2015; Walpola and Hettiarachchi, 2020).

The influence of climate factors can be overcome by selecting genetically modified or adapted strains to different conditions. Trivedi and Sa (2008) successfully obtained mutants of *Pseudomonas corrugata* displaying an increased ability to solubilize phosphate and promote plant growth at lower temperatures. Using an isolation approach, Sarikhani et al. (2019) reported the obtention of thermotolerant *P. agglomerans* strains able to solubilize phosphate at 50°C. A bacterial inoculant based on RP as the carrier was formulated with these *P. agglomerans* strains and displayed stability for 4 months, which is highly convenient for inoculant trade and storage.

Salinity is another factor that influences solubilizing activity. Several studies have successfully selected salt-tolerant PGPB displaying diverse plant benefits under saline stress, including efficient halotolerant PSB and KSB (Jha et al., 2011; Rojas-Tapias et al., 2012; Zhao et al., 2016; He et al., 2018; Ashfaq et al., 2020; Rojas-Solis et al., 2020). Jha and Subramanian (2016) reported KSB belonging to different genera that not only mobilized K from insoluble forms under salinity levels but also protect plants from salinity injury by enhancing their growth-related physiology. Such microorganisms reduced lipid peroxidation and increased plant cell stability under salt stress. Interestingly, Bokhari et al. (2019) isolated several *Bacillus* strains from desert plants but these microorganisms only displayed plant growth promotion ability under salt stress, suggesting that stressful conditions might trigger the production of plant factors that ultimately stimulate yet unknown bacterial factors related to plant tolerance.

The study of plant-bacteria interaction and weathering under extreme environments is useful to prospect weathering strains adapted to these adverse conditions and potentially applicable to agricultural production. Bashan et al. (2002, 2006) reported the presence of rock colonizing plants growing without benefit from the soil, and especially in weathering rocky cliffs, large rocks, and ancient lava flows in hot desert areas of Baja California, Mexico. Then, the same research group evaluated the microorganisms colonizing the rhizoplane of three species of cactus (*Pachycereus pringlei*, *Stenocereus thurberi*, *Opuntia cholla*) and a wild fig tree (*Ficus palmeri*) considered in its previous studies (Puente et al., 2004a,b). The dominant bacterial groups were fluorescent *Pseudomonas* and bacilli. Four isolated bacteria (*Bacillus pumilus* var. 2, *B. subtilis* var. 2, *Actinomadura oligospora*, and *Citrobacter* sp.) were reported to fix N, produce volatile and non-volatile organic acids, and significantly dissolve insoluble P (FePO_4_ and hydroxyapatite), extrusive igneous rock, marble, and limestone. The bacteria were able to release significant amounts of useful minerals, such as P, K, Mg, Mn, Fe, Cu, and Zn, from the rocks. *P. pringlei* seeds inoculated with these bacteria were reported to sprout and grow normally without added nutrients for at least 12 months in pulverized extrusive igneous rock (ancient lava flows) mixed with perlite, while non-inoculated cacti grew less vigorously or died. Several reports describe plant growth promotion of important crops by bacterial strains isolated from deserts and drought regions, including PSB and KSB (Gulati et al., 2010; Kavamura et al., 2013; Alkahtani et al., 2020; Kour et al., 2020b,c). Such microorganisms not only increase nutrient availability but also induce the accumulation of antioxidants and osmolytes, upregulating or downregulating stress-responsive genes (Kour et al., 2020a,b). For more details about the biological weathering by plant and bacteria under desertic and dry conditions, please see the recent review of Lopez and Bacilio (2020).

Another strategy to improve the effectiveness of microbial inoculants based on weathering bacteria is improving the prospection of novel strains displaying a potent mineral solubilizing ability. Traditional methods for isolation and screening of PSB and KSB are frequently based on common media formulations containing insoluble mineral sources. It considers the presence of solubilization haloes produced by bacteria in agar-based screenings or direct detection of soluble P and K released in liquid culture assays (Sharma et al., 2013; Etesami et al., 2017). Bashan et al. (2013a) emphasize that some of these conventional approaches fail to recover effective solubilizing bacteria when using inappropriate mineral sources for PSB screenings. Additionally, most putative PSB were found not to promote plant growth by P solubilization. Soil chemical proprieties are very variable and influence the P dynamics and bacterial solubilizing activity. The identification of effective PSB, for example, requires extensive testing, considering diverse sources of sparingly soluble minerals and plant tests (Bashan et al., 2013b).

Conventional culture media can recover less than 10% of plant-associated bacteria. The introduction of novel methods to increase microbial cultivability presents a huge potential to expand the isolation of plant beneficial organisms (Sarhan et al., 2019). Different methods based on *in situ* and high throughput cultivation have been proposed, such as diffusion chambers, microfluidic streak plate (MSP), hollow-fiber membrane chamber (HFMC), double encapsulation technique, soil substrate membrane system (SSMS), and isolation chip (Ichip) (Sarhan et al., 2019; Lewis et al., 2020). Other approaches include improved growth conditions and development of alternative culture media, especially considering oligotrophs requirements (Vartoukian et al., 2010), once this condition is more effective for the enrichment of rock-weathering bacteria (Hirsch et al., 1995). However, the extent of these methods on the study and recovering of weathering strains agronomically useful should be more explored.

Culture-independent methods based on molecular or genomics approaches can be useful for the identification of new genes and pathways related to mineral solubilization (Alaylar et al., 2020). As mentioned previously, the main characterized genes related to phosphate solubilizing ability are *ppq* and *gcd*. However, despite the frequent report of gluconic acid on the solubilization of minerals by weathering bacteria, several other organic acids display an important role in the weathering process of different minerals (Alori et al., 2017). However, genes, metabolic pathways, and their regulation on the context of weathering remaining poorly characterized.

It is possible to enhance further the biofertilizer potential of bacteria by manipulation of genes related to mineral solubilization (Sashidhar and Podile, 2010). Tripura et al. (2007) used ethyl methanesulfonate mutagenesis to produce *S. marcescens* GPS-5 mutants displaying enhanced mineral solubilizing ability. These authors obtained seven effective mutants showing the maximum (40%) increase in the amount of P solubilized from hydroxyapatite. Using a distinct strategy, Kumar et al. (2013) increased the mineral solubilization capacity of *Enterobacter asburiae* PSI3 by cloning on it an overexpressing *gad* operon derived from *Pseudomonas putida* KT 2440. Such bacteria efficiently produced 2-ketogluconic acid and solubilized RP. Similarly, Farhat et al. (2013) produced an engineered *Escherichia coli* strain expressing the genes *gdh* and *pqq*ABCDE from mineral solubilizing *S. marcescens* CTM 50650. This *E. coli* strain was able to solubilize tricalcium phosphate, hydroxyapatite, and Gafsa rock phosphate (GRP). When immobilized in alginate beads, such microorganisms solubilized the highest amounts of P from GRP under repeated batch fermentation process. Genes related to other organic acids production are also potential targets for genetic engineering. The overexpression of citrate operon in *Herbaspirillum seropedicae* Z67 enhanced the ability to solubilize RP and increased the PGP effect on rice (Wagh et al., 2014).

The understanding of the genetic regulatory mechanism of mineral solubilization can be an important way to improve the efficiency of bacterial weathering. Soluble P has been reported to have an inhibitory effect on P-solubilizing ability and organic acid production by bacteria (Zeng et al., 2016, 2017; Liu J. et al., 2020). Carbon sources (e.g., succinate) commonly present in root exudates could also exhibit a repressive effect on PSB solubilization ability, as demonstrated in *Acinetobacter* sp. SK2 by Bharwad and Rajkumar (2020). These authors discussed that the failure of several PSB in field conditions could be attributed, in part, to these catabolite repression mechanisms, which control the expression of several genes and pathways including that of mineral solubilization. In this sense, regulatory mechanisms of organic acid production and release, as well as other weathering processes, can be a potential target to genetic engineering aiming to increase mineral solubilization and reduce such repressive effects.

The improvement of microbial inoculant formulations is another strategy to enhance the effectiveness of weathering bacteria. When directly applied in soil, PGPB competes with better-adapted indigenous microorganisms, as well as can be predated by soil microbiota, diminishing inoculant efficiency (Bashan, 2016). Inoculant formulations provide a protective microenvironment and increase bacterial viability for long periods, supporting PGPB establishment in soils. Inoculants are a combination of microbial cells with a carrier additive. It is formulated in liquid or solid forms (i.e., peat, powder, and granules) and adapted to different delivery strategies (Bashan et al., 2014). Polymeric substances, especially natural polysaccharides are extensively used as carriers and have demonstrated to increase inoculants efficacy, including for PSB and KSB inoculants. Bacterial encapsulation in a polymer matrix (i.g. alginate) is an advantageous approach that allows a control-release of cells and a higher protective effect compared with other formulations (Chaudhary et al., 2020; Vassilev et al., 2020). Positive effects have been obtained with immobilized PSB in alginate microbeads to increase P uptake by inoculated plants (Schoebitz et al., 2013; Mendoza-Arroyo et al., 2020). However, the widespread adoption of this technique has been limited by its high cost (John et al., 2011). The introduction of innovative materials as carriers is a promising tactic to improve inoculant formulations for mineral solubilization. Recently, Safari et al. (2020) employed nanomaterials (nanoclay and natural char nanoparticles) as addictive in PSB inoculants. Such formulations based on *P. putida* (PP20) and *Pseudomonas kilonensis* (PK11) strains successfully maintained bacterial viability and efficacy in solubilizing phosphate. In addition to improved formulations, the use of atmospheric pressure non-thermal plasma has a great potential to increase the vitality of PGPB. Ji et al. (2019) applied this technique in *B. subtilis* CB-R05, and such treatment accelerated bacterial growth and motility, increased bacterial colonization of plants, consequently enhancing plant growth-promoting effects.

Most of the studies discussed in the previous topics deal with a single bacterial inoculant or a combination of few microorganisms. However, the weathering process in soil occurs by integrative metabolism of plenty of microorganisms distributed on soil, each one possibly displaying particular weathering abilities (Wang et al., 2017). A better understanding of microbial weathering requires an integrative investigation of microbial communities and their complex interaction with different abiotic factors. Uroz et al. (2015) suggest that the environment on the surface and surround rocks particles and soil minerals composes a specific habitat of micro-organisms called “mineralosphere.” Such a region is an inorganic analogous to the rhizosphere. Mineralosphere is affected by external factors such as soil pH, temperature, and moisture, but also directly control the bacterial diversity by intrinsic characteristics such as mineral chemistry, weatherability, surface structure, and porosity (Uroz et al., 2015). The use of rock powders as fertilizers associated with bacteria requires considering the different mechanisms/factors that direct both environments, mineralosphere, and rhizosphere, and how these factors connect both regions. Several culture-independent methods, mainly based on metagenomic approaches, have expanded the knowledge about the dynamics of soil microbial communities under weathering and crop production (Chhabra et al., 2013; Uroz et al., 2013; Carbonetto et al., 2014; Gómez-Merino et al., 2015).

Microbial inoculants can lead to significant changes in the diversity and abundance of indigenous soil microbial communities affecting their functional capabilities. Inoculated bacteria establish antagonistic/synergistic interactions with different members of the microbial rhizosphere community and modulate differently the plant responses (Trabelsi and Mhamdi, 2013). Bacterial inoculants could alter even the protist community structure. This is an important aspect in plant-bacteria interaction, once these microbial eukaryotic groups are key predators in soil and directly affect the performance of desirable microorganisms, such as PGPB (Xiong et al., 2019). In the context of mineral solubilization for crop nutrition, especially related to P solubilization, some studies described the effect of applying rock fertilizers or solubilizing strains on plant microbiota. P fertilization regimes can be the dominant factor driving bacterial community structure in soil (Wang et al., 2018). The long-term-fertilization with RP has been associated with a higher selection of P-solubilizing community in the rhizosphere than the observed effect of high soluble P fertilizers (Silva et al., 2017). The inoculation of a PSB could not lead the strain to not become predominant in the soil community; however, it can significantly change the abundance of other taxonomical groups in the rhizosphere (Liu Y.-Q. et al., 2020). Access plant-associated microbiomes structure and their functions is very strategic in an agronomical context since such microbial communities can be modulated to achieve a higher yield and a sustainable production (Andreote and Pereira E Silva, 2017). This is especially important considering global warming and how the microbiota is reacting and affecting crop responses to climate change (Dubey et al., 2019).

A specific function associated with community members from a complex microbiome can be achieved by a “deconstructing” approach. Using specific defined media and enrichment cultivation techniques direct to a functional target, an initial soil microbiota can be reduced to a low-complexity and specialized community that can be particularly studied and explored (Naylor et al., 2020). The microbiota can be directly engineered to perform a desirable function, and different approaches have been developed to achieve this including alternatives based on genetic manipulation of the microbial community (Lawson et al., 2019). Synthetic biology strategies are very promising to microbiota engineering and can be performed under a bottom-up or top-down approach. In a bottom-up approach, selected bacterial strains from the natural community can be genetically modified to carry a desirable trait (i.e., P or K solubilizing ability) and used to assemble a synthetic microbial community. In the top-down approach, desirable traits can be introduced into a range of microbial hosts *in situ* by gene horizontal transfer using an engineered conjugative donor strain or by bacteriophages (Ke et al., 2020). In the concern of bacterial weathering and rock fertilizers, rational microbiome design could be used to increase weathering traits in soil microbial communities or to introduce other PGP traits and genes for plant colonization/interaction into efficient soil weathering microorganisms.

## Final Remarks

In the present review, we addressed rock fertilizers. They are inexpensive and environmentally friendly options for farmers. We focused on biological modification processes driven by microbial activity to improve the agronomic effectiveness of these non-conventional nutrient-bearing rock and mineral resources as fertilizers. We found a series of reports demonstrating that there is considerable potential in the combination of inoculants with rock fertilizers to increase soil fertility and crop production. We consider important to further expand our understanding of this alternative approach, in particular by providing consistent answers to key questions such as (i) what is the best inoculation rock/mineral combination for each crop? (ii) what are the parameters affecting inoculant efficacy on nutrient release from rocks and mineral fertilizers? (iii) how to maximize the weathering activity of microbial inoculants to meet the crop’s nutritional demands? and (iv) how to adapt inoculation methods and strategies to local needs considering environmental variations and socioeconomic factors?

The integrated application of microbial inoculants and rock fertilizers requires the efforts of different areas and specialists. The choice for rock resources and bacterial strains demands rigorous field-scale trials to validate agronomic effectiveness. Similarly, the extension of environmental benefits resulting from such practice must be precise and the risks must be estimated, ensuring security, productivity, and sustainability.

## Author Contributions

LV, CG, and BL elaborated the conception of the study. IR and CV wrote the manuscript. LV, CG, BL, and LP revised the manuscript critically. All authors read and approved the final manuscript.

## Conflict of Interest

The authors declare that the research was conducted in the absence of any commercial or financial relationships that could be construed as a potential conflict of interest.
